# Supplementary material for: Rolling the evolutionary dice: Neisseria commensals as proxies for elucidating the underpinnings of antibiotic resistance mechanisms and evolution in human pathogens
Source: bioRxiv. 2023 Sep 26:2023.09.26.559611. Preprint. [Version 1] doi: 10.1101/2023.09.26.559611 (PMC10557713; doi:10.1101/2023.09.26.559611)
Supplement: Supplement 1 [file media-1.pdf]

Supplemental Table 2. The impact of species on evolved MICs, 2-way ANOVA interaction results ordered by adjusted P-value

Comparision	Adjusted P
#AZI:N.elongata-AZI:N.cinerea	0.03599
#AZI:N.subflava-AZI:N.elongata	0.03599
#AZI:N.cinerea-AZI:N.canis	0.4938929
#AZI:N.subflava-AZI:N.canis	0.4938929
#AZI:N.elongata-AZI:N.canis	0.8257135
#PEN:N.cinerea-PEN:N.canis	0.9999931
#PEN:N.elongata-PEN:N.cinerea	0.9999968
#PEN:N.subflava-PEN:N.cinerea	0.9999995
#PEN:N.elongata-PEN:N.canis	1
#PEN:N.subflava-PEN:N.canis	1
#AZI:N.subflava-AZI:N.cinerea	1
#PEN:N.subflava-PEN:N.elongata	1

Supplemental Table 3. The impact of species on MIC fold-change, 2-way ANOVA interaction results ordered by adjusted P-value

Comparision	Adjusted P
#AZI:N.elongata-AZI:N.canis	0.0000018
#PEN:N.cinerea-PEN:N.canis	0.0001473
#AZI:N.subflava-AZI:N.elongata	0.0002319
#PEN:N.subflava-PEN:N.cinerea	0.0045885
#AZI:N.cinerea-AZI:N.canis	0.0084122
#PEN:N.elongata-PEN:N.cinerea	0.0085094
#AZI:N.elongata-AZI:N.cinerea	0.0300176
#AZI:N.subflava-AZI:N.canis	0.4613159
#AZI:N.subflava-AZI:N.cinerea	0.4870153
#PEN:N.elongata-PEN:N.canis	0.7070757
#PEN:N.subflava-PEN:N.canis	0.8444889
#PEN:N.subflava-PEN:N.elongata	0.9999949
