# Supplementary material for: Rolling the evolutionary dice: Neisseria commensals as proxies for elucidating the underpinnings of antibiotic resistance mechanisms and evolution in human pathogens
Source: bioRxiv. 2023 Sep 26:2023.09.26.559611. Preprint. [Version 1] doi: 10.1101/2023.09.26.559611 (PMC10557713; doi:10.1101/2023.09.26.559611)
Supplement: Supplement 2 [file media-2.pdf]

Supplementary Table 1. MICs and number of derived mutations compared to ancestral reference sequences for single colony picks of evolved lineages

Species	Strain	Selective agent	MIC of derived strains after selection
N. canis	AR0948.C1	Azi	0.38
N. canis	AR0948.C2	Azi	0.38
N. canis	AR0948.C3	Azi	0.38
N. canis	AR0948A.1	Azi	16
N. canis	AR0948B.1	Azi	48
N. canis	AR0948C.1	Azi	48
N. canis	AR0948D.1	Azi	32
N. canis	AR0948.C1	Pen	0.25
N. canis	AR0948.C2	Pen	0.25
N. canis	AR0948.C3	Pen	0.25
N. canis	G4.S1.1	Pen	3
N. canis	G4.S2.1	Pen	†
N. canis	G4.S3.2	Pen	2
N. canis	G4.S4.1	Pen	†
N. cinerea	AR0944.C1	Azi	8
N. cinerea	AR0944.C2	Azi	6
N. cinerea	AR0944.C3	Azi	8
N. cinerea	G2.S1.1	Azi	†
N. cinerea	G2.S2.1	Azi	256
N. cinerea	G2.S3.1	Azi	†
N. cinerea	G2.S4.1	Azi	256
N. cinerea	AR0944.C1	Pen	0.38
N. cinerea	AR0944.C2	Pen	0.38
N. cinerea	AR0944.C3	Pen	0.38
N. cinerea	AR0944A.1	Pen	12
N. cinerea	AR0944B.1	Pen	4
N. cinerea	AR0944C.1	Pen	2
N. cinerea	AR0944D.1	Pen	6
N. elongata	AR0945.C1	Azi	0.5
N. elongata	AR0945.C2	Azi	0.75
N. elongata	AR0945.C3	Azi	0.5
N. elongata	AR0945A.1	Azi	†
N. elongata	AR0945B.1	Azi	1
N. elongata	AR0945C.1	Azi	0.5
N. elongata	AR0945D.1	Azi	0.25
N. elongata	AR0945.C1	Pen	0.25
N. elongata	AR0945.C2	Pen	0.25
N. elongata	AR0945.C3	Pen	0.25
N. elongata	G5.S1.1	Pen	1
N. elongata	G5.S2.1	Pen	†
N. elongata	G5.S3.1	Pen	2
N. elongata	G5.S4.1	Pen	12
N. subflava	G1.S1.1	Azi	256
N. subflava	G1.S2.1	Azi	256
N. subflava	G1.S3.1	Azi	96
N. subflava	G1.S4.1	Azi	96
N. subflava	G3.S1.1	Pen	4
N. subflava	G3.S2.1	Pen	6
N. subflava	G3.S3.1	Pen	4
N. subflava	G3.S4.1	Pen	0.75
N.subflava	AR0953.C1	Azi	2
N.subflava	AR0953.C2	Azi	1
N.subflava	AR0953.C3	Azi	1
N.subflava	AR0957.C1	Azi	8
N.subflava	AR0957.C2	Azi	8
N.subflava	AR0957.C3	Azi	8
N.subflava	AR0953.C1	Pen	1
N.subflava	AR0953.C2	Pen	1.5
N.subflava	AR0953.C3	Pen	1.5
N.subflava	AR0957.C1	Pen	1
N.subflava	AR0957.C2	Pen	1
N.subflava	AR0957.C3	Pen	1

† Stocks of single colony picks did not grow after the initial patch plate
